# Anesthesia and analgesia for common research models of adult mice

**DOI:** 10.1186/s42826-022-00150-3

**Published:** 2022-12-13

**Authors:** Siavash Ahmadi-Noorbakhsh, Mohammad Farajli Abbasi, Maedeh Ghasemi, Gholamreza Bayat, Nahid Davoodian, Ehsan Sharif-Paghaleh, Seyedeh Mahsa Poormoosavi, Melika Rafizadeh, Maryam Maleki, Hesamaddin Shirzad-Aski, Hossein Kargar Jahromi, Masoomeh Dadkhah, Bahman Khalvati, Tahereh Safari, Mohammad Amin Behmanesh, Seyed Esmaeil Khoshnam, Gholamreza Houshmand, Sayyed Alireza Talaei

**Affiliations:** 1 Preclinical Core Facility (TPCF), Tehran University of Medical Sciences, Tehran, Iran; 2 The National Ethics Committee for Biomedical Research, Floor 13th, Complex A, Ministry of Health and Medical Education, Eyvanak Blvd., Shahrake Gharb, Tehran, Iran; 3 Neuroscience Research Center, Institute of Neuropharmacology, Kerman University of Medical Sciences, Kerman, Iran; 4 Department of Physiology, School of Medicine, Isfahan University of Medical Sciences, Isfahan, Iran; 5 Department of Physiology-Pharmacology-Medical Physic, School of Medicine, Alborz University of Medical Sciences, Karaj, Iran; 6 Endocrinology and Metabolism Research Center, Hormozgan University of Medical Sciences, Bandar Abbas, Iran; 7 Department of Immunology, School of Medicine, Tehran University of Medical Sciences, Tehran, Iran; 8 Department of Imaging Chemistry and Biology, School of Biomedical Engineering and Imaging Sciences, Faculty of Life Sciences and Medicine, King’s College London, London, England; 9 Department of Histology, School of Medicine, Research and Clinical Center for Infertility, Dezful University of Medical Sciences, Dezful, Iran; 10 Department of Pharmacology, Medical School, Shahid Beheshti University of Medical Sciences, Tehran, Iran; 11 Department of Physiology, Faculty of Medicine, Ilam University of Medical Sciences, Ilam, Iran; 12 Infectious Diseases Research Center, Golestan University of Medical Sciences, Gorgan, Iran; 13 Research Center for Non-Communicable Disease, Jahrom University of Medical Sciences, Jahrom, Iran; 14 Pharmaceutical Sciences Research Center, Ardabil University of Medical Sciences, Ardabil, Iran; 15 Medicinal Plants Research Center, Yasuj University of Medical Sciences, Yasuj, Iran; 16 School of Medicine, Department of Physiology, PhD, Zahedan University of Medical Sciences, Zahedan, Iran; 17 Pharmacology Research Center, Zahedan University of Medical Sciences, Zahedan, Iran; 18 Department of Histology, School of Medicine, Dezful University of Medical Sciences, Dezful, Iran; 19 Medical Basic Sciences Research Institute, Ahvaz Jundishapur University of Medical Sciences, Ahvaz, Iran; 20 Psychiatry and Behavioral Sciences Research Center, Addiction Institute, Department of Pharmacology, Faculty of Medicine, Mazandaran University of Medical Sciences, Sari, Iran; 21 Physiology Research Center, Institute for Basic Sciences, Kashan University of Medical Sciences, Kashan, Iran

**Keywords:** Anesthetic, Analgesic, Mouse, Pharmacology, Side effect, Preclinical

## Abstract

Anesthesia and analgesia are major components of many interventional studies on laboratory animals. However, various studies have shown improper reporting or use of anesthetics/analgesics in research proposals and published articles. In many cases, it seems “anesthesia” and “analgesia” are used interchangeably, while they are referring to two different concepts. Not only this is an unethical practice, but also it may be one of the reasons for the proven suboptimal quality of many animal researches. This is a widespread problem among investigations on various species of animals. However, it could be imagined that it may be more prevalent for the most common species of laboratory animals, such as the laboratory mice. In this review, proper anesthetic/analgesic methods for routine procedures on laboratory mice are discussed. We considered the available literature and critically reviewed their anesthetic/analgesic methods. Detailed dosing and pharmacological information for the relevant drugs are provided and some of the drugs’ side effects are discussed. This paper provides the necessary data for an informed choice of anesthetic/analgesic methods in some routine procedures on laboratory mice.

**Supplementary Information:**

The online version contains supplementary material available at 10.1186/s42826-022-00150-3.

## Background

Anesthesia and analgesia (A&A) are the main components of many procedures on laboratory animals. However, studies suggest that a considerable number of researches are based on improper use or reporting of A&A methods [1, 2]. In a study on 400 articles that included surgical models in mouse and other species, the authors concluded that the animal A&A are not reliably presented in the literature [3]. Another study also shows mouse research is essentially not reported well in the European literature [4].

Part of this problem may be related to insufficient training of investigators regarding A&A in laboratory animals [2]. For example, there is a common myth among some researchers assuming anesthesia is equivalent to analgesia. Accordingly, some papers may report using a hypnotic drug (pentobarbital) without analgesia for performing painful recovery surgeries on mice [5]. Navarro et al., discuss that the current method of peer-to-peer training in laboratory animal science, may causes misinterpretation of A&A concepts among researchers [6]. Also, the unfamiliarity of researchers with available anesthetic options in mice has led to limited range of anesthetic protocols in this animal [6].

Another part of this problem may be related to responses of A&A to specific anatomic and physiologic attributes of mice. For example, fast metabolism and excretion of A&A drugs in mice, reduces the half-life of these drugs and limits the duration of A&A. This along with reduced liver glycogen reserve in mice, promote hypoglycemia. When compared with larger species, the higher ratio of body surface area to body volume in mice leads to faster heat loss and hypothermia [7]. Small body size of mice also causes physical problmes. For example, endotracheal intubation and ventilation of this species are demanding tasks, and many veterinary anesthesia monitoring devices could not be used for mice anesthesia.

There may be other explanations for this large number of inappropriate A&A methods, such as fewer regulations in animal research (in comparison to veterinary clinical practice), lower availability of anesthetic monitoring devices in research settings, or high diversity of research procedures requiring anesthesia.

It is while, proper induction and maintenance of A&A are essential elements of maintaining animal welfare and preserving the scientific integrity of research [6, 8]. In one hand, distress and pain are components of many experimental procedures and substantially affect animal welfare. Therefore, proper use of sedatives, tranquilizers, analgesics and anesthetics are essential ethical requisites of many experiments [7]. On the other hand, using these drugs has two types of effects: (1) Alleviating the distress and pain of the animal which have important biochemical and physiologic effects on samples and data generated by the animal. For example, it is shown that pain causes an increase of epinephrine, cortisol, plasma glucose, endorphins, and many other chemicals in the body. Pain also increases heart rate, stroke volume, cardiac output, and leads to shallow fast breathing, etc. [9]. Proper use of analgesics can substantially prevent these effects and produce good data; (2) Direct physiologic effect of the drug on the animal and its corresponding samples and data [10]. For example, isoflurane better preserve cardiovascular functions than halothane. However, it is a potent suppressor of the respiratory system and its vasodilatory effects are more than halothane [11]. Therefore, proper choose of drugs in experiments can minimize the side effects of the drugs on research data.

The laboratory mouse is the most used species of animals in research, with an estimate of 61% of laboratory animals used in the European Union in 2017 [12]. Therefore, A&A in mice may constitute a major part of the problem with laboratory animal anesthesia. There are at least three invaluable papers [6, 7, 13] with a detailed discussion of A&A in mice. However, these papers have mainly discussed A&A methods according to the classes of medications rather than the actual procedures. Although this may be helpful in some circumstances, researchers are usually encountered with the question of “which anesthetic protocol should I use for procedure X?”. In this review, we would like to answer this type of question by discussing A&A for routine procedures on adult mice. We used appropriate references in this regard and critically appraised the quality of their anesthetic methods. The dose of anesthetic and perianesthetic medications are presented in Additional file 1. Analgesics doses are presented in Additional file 2. Important pharmacological data for most common anesthetic agents are presented in Additional file 3. An overview of the anesthetic modalities for various procedures on adult laboratory mice is provided in Table 1.

**Table 1:** Suggested anesthetics for common procedures on adult mice. For details, please refer to the text

Procedure/body organ	Suggested anesthesia/modality
Abdominal aorta	Isoflurane AND buprenorphine AND postoperative analgesia: buprenorphine
Agitated animals	Low dose acepromazine
Brain	(Isoflurane OR ketamine/xylazine) AND buprenorphine AND (subcutaneous lidocaine with or without bupivacaine AND splash block of periosteum with lidocaine) AND dexamethasone (in craniotomies) AND postoperative analgesia: buprenorphine
Gastrointestinal tract	{Isoflurane OR sevoflurane OR ketamine/xylazine/acepromazine OR pentobarbital (not for models of inflammatory bowel disease)} AND buprenorphine AND postoperative analgesia: buprenorphine
Imaging	Restraint OR Ketamine/xylazine OR pentobarbital OR isoflurane OR sevoflurane OR desflurane
Immobilization (long-term) for non-painful procedures	Hypnotics (e.g., thiopental or pentobarbital)
Immobilization (temporary) for non-painful procedures	Higher dose of acepromazine OR flash anesthetic techniques using an anesthetic chamber (isoflurane or halothane)
Injections and catheterizations	Animal training OR distraction OR lidocaine-prilocaine OR flash anesthesia using an isoflurane chamber
Kidneys	(Ketamine/xylazine OR ketamine/medetomidine OR ketamine/xylazine/acepromazine) AND buprenorphin﻿e. For renal ischemia model: pentobarbital AND buprenorphine. Postoperative analgesia: buprenorphine
Laparotomy	(Isoflurane OR sevoflurane OR ketamine/xylazine/acepromazine) AND lidocaine line block with or without bupivacaine at the intended site for incision AND postoperative analgesia: buprenorphine
Limbs	{Isoflurane AND dinitrogen monoxide AND oxygen AND opioids (e.g., buprenorphine or morphine) AND NSAIDs (e.g., ketoprofen or flunixin meglumine)} OR {ketamine/xylazine AND sustained-release buprenorphine} OR {isoflurane AND buprenorphine AND lidocaine splash block} OR {midazolam/medetomidine} OR {fentanyl/midazolam} AND postoperative analgesia: buprenorphine
Liver and bile duct	{Isoflurane OR (isoflurane AND meloxicam AND buprenorphine) OR sevoflurane OR ketamine/xylazine/acepromazine} AND postoperative analgesia: buprenorphine
Ophthalmic procedures	{Oxybuprocaine eye ointment OR (ketamine/medetomidine; postoperative analgesia: meloxicam) OR (isoflurane AND 0.5% proparacaine hydrochloride ophthalmic solution} AND glycopyrrolate AND postoperative analgesia
Ovaries	{Isoflurane OR halothane OR (ketamine/medetomidine)} AND buprenorphine AND ketoprofen
Sepsis	Surgical models: (isoflurane OR ketamine/xylazine) AND (lidocaine with or without bupivacaine) AND buprenorphine
Spinal cord	{Isoflurane AND buprenorphine AND local analgesia (in laminectomy and crushing of the spinal cord)} OR Ketamine/xylazine (in neural stem cells transplantation) AND postoperative analgesia
Stroke	Isoflurane AND buprenorphine
Thoracotomy	Please refer to the text
Tracheostomy	Depending on the main surgical procedure; fentanyl/dexmedetomidine/midazolam OR (pentobarbital sodium/ketamine OR ketamine/xylazine OR isoflurane OR pentobarbital/Ketamine/xylazine OR propofol) AND buprenorphine AND postoperative analgesia
Wound models	(Ketamine/xylazine OR medetomidine/midazolam/butorphanol OR isoflurane/buprenorphine) AND postoperative analgesia: systemic or topical opioids, NSAIDs, local anesthetics, tramadol local infiltration

## Anesthesia

Anesthesia may be divided into three different concepts: local anesthesia, general anesthesia, and surgical anesthesia. Local anesthesia (aka local analgesia) refers to the loss of pain sensation in a specific body area [10]. General anesthesia is a drug-induced state of reversible unconsciousness, in which the animal could not be aroused by noxious stimuli [10] but it does not necessarily mean that the animal does not sense or perceive pain. Surgical anesthesia is a state of general anesthesia that has four components: unconsciousness, amnesia, muscular relaxation, and analgesia [10]. Currently, no anesthetic agent exists that could be solely used for achieving surgical anesthesia. Therefore, various agents are used to develop various components of surgical anesthesia and this is called ‘balanced anesthesia’. In a research setting, the method of anesthesia for mice may be selected according to the duration and type of the procedure, comfortability of the anesthetist with a method, and availability of required drugs and devices. Understanding the effects of anesthesia on research outcomes may determine the selection of the anesthetic method. When there is no alternative method available, this knowledge helps in inferring the achieved results. For example, some studies have reported that pentobarbital sodium or isoflurane may significantly alter tissue compositions [14–19]. Also, it is shown that short-term (2 min) and long-term (more than 10 min) anesthesia with either pentobarbital sodium or isoflurane before transcardial perfusion can make alterations in brain immunohistochemistry results [20].

Surgical anesthesia may be divided into five parts: (1) pre-anesthetic preparation including the selection of an anesthetic candidate and its preparation for anesthesia/surgery; (2) anesthetic induction, as the process by which a conscious animal is rendered unconscious; (3) anesthetic maintenance, that is maintaining an animal unconscious throughout the procedure; (4) anesthetic recovery, as the process by which an unconscious animal regains consciousness; and (5) post-anesthetic care, which includes animal care to gain full consciousness.

## Analgesia

Surgical pain feeling includes nociception and nociperception. Nociception comprises the encoding of noxious stimuli to neural impulses (transduction), transmitting the encoded neural impulses to the CNS (transmission), and modulating neural impulses before delivering them to the brain (modulation). Nociception is independent of CNS activity [10]. In contrast, nociperception is the translation of neural impulses to conscious perception of pain in the brain [10].

Accordingly, analgesia refers to methods of inhibiting or interrupting nociception or nociperception, so that the patient does not feel pain in response to noxious stimuli that are otherwise painful [10]. Although general anesthesia may inhibit ‘nociperception’ to varying extents, it generally does not affect nociception. Therefore, if effective analgesic methods are not used, we may have an animal under general anesthesia but with ongoing nociception. Furthermore, anesthetic drugs may suppress motor functions and physical signs related to pain. At lower levels of unconsciousness (i.e. subanesthesic doses), the animal may perceive pain but may be unable to demonstrate associated physical signs. This may occur during the anesthetic recovery of an animal that has not received proper perioperative analgesia.

## Pre-anesthetic preparation

Pre-anesthetic preparation of mice is best described elsewhere [6, 7]. Anticholinergics (atropine or glycopyrrolate) may be used to reduce salivary and bronchial secretions and avoid the substantial drop in heart rate due to anesthetic medications or surgical manipulations [7]. Anesthesia can be induced less than 10 min following IP administration of atropine.

## Anesthetic monitoring and perianesthetic care

Anesthetic monitoring and perianesthetic care are best discussed in previous studies [6, 7, 13]. Briefly, the following parameters should be monitored and corrected if needed: depth of anesthesia, analgesia (hypoalgesia), body temperature, blood glucose level, body hydration, respiratory status (depth, rate, and pattern), heart and vascular functions (heart rate, capillary refill time, mucus membranes’ color, and pulse quality) and blood oxygen level (pulse-oximetry). The cornea should be kept moistened (e.g., by vitamin A + D ophthalmic ointment or wet pads). The animal should be protected from postural hypotension.

## Induction

When using injectable anesthetics in mice, induction is usually indistinguishable from maintenance. This is in contrast to inhalational anesthesia, which usually includes a separate induction phase. We will discuss the induction along with maintenance, in the following sections.

## Maintenance

### Injections and catheterizations

Simple injections may induce a sudden sharp pain in animals. This may have stress-related consequences that may confound the outcomes of some studies. To reduce this effect, training of the animal may be considered before using them in projects [21], and this is a proven method of desensitizing the animal to simple injections. This is well demonstrated by the RISE institute (www.ri.se). Also, one may distract the animal by talking, giving treats, or gently touching its body while the injection is given.

To completely eliminate the pain, the topical anesthetic compound lidocaine-prilocaine (EMLA® cream) can be applied on the skin, 30–60 min before needle insertion [22, 23]. Flash anesthesia using an isoflurane chamber is suggested for inducing anesthesia for catheterization with minimal hemodynamic effects [24–26]. However, we think that the stress of anesthesia (even very quickly) is more than the stress induced by the insertion of a hypodermic needle.

### Working with agitated animals

Performing procedures on agitated animals may require tranquilization with a lower dose of acepromazine. It should be noted that the animal can still perceive pain in this condition [27, 28].

### Temporary immobilization for non-painful procedures

For some non-painful procedures, the animal may require a higher level of sedation. This may be useful for imaging techniques that require temporary (~ 5 min) immobilization. It may be accomplished in mice by using a higher dose of acepromazine. Flash anesthetic techniques using an anesthetic chamber (isoflurane or halothane), may also produce a sleep-like state in mice. To extend the duration of anesthesia, the nose cone method [29] can be used.

### Longer immobilization for non-painful procedures

This may be used for long imaging processes or any non-painful procedure requiring complete immobilization. In this regard, hypnotics such as thiopental or pentobarbital may be used.

### Imaging

In the majority of the imaging technologies, the mice need to be as calm as possible since movements could lead to disruption of signal acquisition and this would lead to false data generation [30]. The mice may be immobilized with restraining or anesthetizing. For some studies where anesthesia would interfere with the metabolic and biochemical processes of the study, the mouse should be restrained and imaged [31]. There are studies carried out on imaging devices where the animal would be free and imaged at the same time. This requires a device to be surgically attached to the animal so that the signals would be acquired [32].

Restraining a mouse during the imaging acquisition for a duration of seconds to hours is stressful and adds confounding factors to the imaging process. Therefore, if possible, the mouse should be anesthetized. Since most imaging procedures do not incur pain, analgesia may not be required [33]. However, for invasive imaging techniques, the type of analgesia is determined according to the type and extent of the invasive procedure.

In a rat study, it was shown that ketamine and chloral hydrate increase the binding of receptor ligands to dopamine D1 receptors in Positron emission tomography (PET) imaging [34], whereas pentobarbital decreased the binding. Similar findings are expected in mice. Ketamine and xylazine also were shown to increase the blood fluorodeoxyglucose (FDG) activity and reduce the tumor FDG uptake, causing a less contrast image to be acquired using PET imaging [35]. The same study also demonstrated the same effect using pentobarbital, but its effect was less prominent than ketamine, and xylazine. Isoflurane, sevoflurane, and desflurane inhibit the activity of the luciferase enzyme, which generates signals for detection by bioluminescence imaging [36]. Also, repetitive sevoflurane usage has an immunomodulatory effect in mice and this could affect the radiation imaging and the well-being of the animal [37]. Isoflurane was also shown to reduce the uptake of radioactively labeled FDG and subsequently PET imaging in mouse heart and brain, due to overall hypothermia caused by isoflurane [38]. Respiratory acidosis, as a complication of anesthesia, is concurrent with an increase in blood pCO_2_ level and this may affect PET imaging with FDG, in mouse models of cancer and arthritis [39]. More details on the anesthetic methods for imaging are presented elsewhere [13, 40].

### Ophthalmic procedures

Heat, mechanical, and chemical stimuli could cause neurological reactions in most corneal nerves. Topical anesthetics may be used to reversibly block sodium channels of corneal nerves and avoid the transfer of impulses in the cornea, conjunctiva, and sclera [41]. Oxybuprocaine eye ointment may be used to provide local analgesia for ophthalmic procedures [7].

For enucleation, researchers have used intraperitoneal ketamine-medetomidine. At the end of the surgery, the effect of medetomidine was reversed using intraperitoneal atipamezole. Meloxicam was used as a postoperative analgesia [42]. It is shown that medetomidine could reduce tear production in a dose-dependent fashion [43]. It should be noted that administration of ketamine-medetomidine could not provide surgical anesthesia consistently. Therefore, anesthetic depth monitoring should be continuously performed before and during surgical procedures [44] and analgesics (such as buprenorphine) should be used when the anesthetic depth is insufficient. This combination is generally advised for minor procedures on mice [45].

For intravitreal implant injection, the mice were anesthetized using ketamine-xylazine [46]. This combination has also been used for developing an ocular hypertension model [47]. However, this combination is shown to induce corneal damage, irrespective of the treatments applied, to prevent corneal dehydration [48]. Ketamine-xylazine combination is believed to alter the ocular physiology and cause xylazine-mediated reversible cataracts [49, 50]. This combination may also cause retinal Müller cell reactivity [49].

For performing retro-orbital injection to the venous sinus, anesthesia was induced using an isoflurane induction chamber [51]. Then, the mouse was connected to a non-rebreathing anesthetic system via a funnel-shaped nose cone. A drop of 0.5% proparacaine hydrochloride ophthalmic solution was applied to the eye to provide sufficient analgesia for a painless procedure [51]. It should be noted that blood sampling from retro-orbital venous sinus is considered a terminal procedure in some countries (e.g., UK) and the animal should be euthanized after the procedure without gaining consciousness.

Intraperitoneal injections of a diluted solution of Hypnorm™ (fentanyl/fluanisone) and midazolam have been used for inducing conjunctival scarring models [52, 53]. In rats, this combination could also cause histologic corneal lesions with scores of 2–4. The severity and incidence rate of corneal lesions when using Hypnorm™ and midazolam in rats is less than ketamine-xylazine but more than pentobarbital or isoflurane [54]. Midazolam inhibits Vascular endothelial growth factor-mediated events in retinal cells, thus preventing hyperglycemia-induced vascular leakage in a mouse model of diabetes [55].

Various methods of anesthesia could affect the intraocular pressure (IOP) and this requires IOP monitoring before and during anesthesia. For example, anesthesia with ketamine/xylazine may result in an increased IOP [41, 56]. Ketamine/xylazine can also cause hyperopia and change the refraction of the eye. However, it is shown that pentobarbital has no effect on eye refraction [57]. To prevent oculocardiac reflex resulting from eyes manipulation, one may use atropine or glycopyrrolate [58].

### Limbs

Limb procedures include limb ischemia model, model of gangrene, ischemia–reperfusion models, lymphedema, heat-induced limb length asymmetry, nerve transection, and limb transplantation. There are three types of pain involved in models involving femoral artery ligation (e.g. limb ischemia, gangrene, or ischemia–reperfusion models): (1) a somatic pain resulting from skin incision, subcutaneous tissue dissection, and skin suturing; and (2) a neurogenic pain caused by femoral nerve manipulation, and (3) ischemic pain consequent to tissue hypoxia caused by arterial ligation.

For the limb ischemia model, general anesthesia comprising 1.0 to 1.5% isoflurane, 60% dinitrogen monoxide, and 40% oxygen were used [59]. However, this anesthetic regimen seems to lack the potent analgesic component of balanced anesthesia. Therefore, we would add proper analgesics such as opioids (e.g., buprenorphine or morphine) and nonsteroidal anti-inflammatory drugs (NSAIDs; e.g., ketoprofen or flunixin meglumine) to this anesthetic regimen to provide surgical anesthesia. General anesthesia with an intraperitoneal injection of ketamine/xylazine is reported in the literature [60, 61] and seems to be more pertinent to this operation. Another study [62] has concluded that isoflurane is the most used inhalational anesthetic compound for modeling limb ischemia in mice. It has also ranked a combination of midazolam/medetomidine to be one of the most used injectable anesthetic compounds for this model [62]. Because of the early peripheral vasoconstriction induced by alpha-2 agonists, such as xylazine, the use of these agents in research concerning vessels with large amounts of vascular smooth muscles, is argued [62].

In a model of gangrene, intraperitoneal ketamine/xylazine accompanied by subcutaneous injection of sustained-release buprenorphine was used for performing ligation surgery on the femoral artery and vein [63].

For the ischemia–reperfusion model using the open and closed techniques, mice were anesthetized by inhalational isoflurane and subcutaneous buprenorphine [64].

Lymphedema models include irradiation with a single dose of 30 Gy ionizing radiation, using an X-ray machine. For this purpose, intraperitoneal injection of pentobarbital [65] or inhalational isoflurane [66] is used. After one week, a surgical operation was done under isoflurane anesthesia [65]. The 90-min surgical operation included a subcutaneous injection of a dye in the left paw, skin incision at the left inguinal region, ligature of the lymphatic vessels at three points, resection of the subiliac and popliteal lymph nodes, and suturing of the skin to its underlying muscles without apposing the skin edges [65]. Accordingly, reported isoflurane anesthesia lacks the analgesic component. We suggest adding an opioid analgesic (e.g., buprenorphine) to this protocol. As an alternative to isoflurane, another study has used subcutaneous fentanyl/midazolam for this surgery [66]. Postoperative analgesia included subcutaneous buprenorphine every 8 h [65].

In a model of heat-induced limb length asymmetry, the right sides of the body of the mice were positioned on a heating pad with a 40 °C constant temperature. For this purpose, isoflurane inhalation was used to hold the mice in lateral recumbency [67]. For Laser Doppler perfusion imaging of limbs, mice were lightly anesthetized by first inducing anesthesia in an induction chamber, and then by maintaining anesthesia by a nasal cannula delivering isoflurane/oxygen [63].Nerve transection on limbs may be performed on sciatic or femoral nerves. For this purpose, anesthesia was induced with 2–3% isoflurane. A single dose of buprenorphine was administered “prior to starting the surgery” [68]. In this regard, we suggest buprenorphine injection be performed about 1 h before starting the first surgical incision so that the preemptive analgesic requirement is met. We would also suggest a splash block with a drop of 1% lidocaine at the nerve resection site.

For limb transplantation research, limb resection is performed on a donor and recipient by incising the skin, muscles, nerves, and vascular structures and mid-shaft femoral transection [69]. The resected limb is then implanted in the recipient, by suturing appropriate structures together. For this purpose, analgesia was achieved using buprenorphine, administrated 1 h before the first skin incision. Anesthesia was induced in an isoflurane induction chamber and maintained by a nose cone delivering isoflurane and oxygen [69]. Since the procedure involves both soft tissues and hard tissue transections, we suggest using a lidocaine splash block on the nerves and also the femoral shaft before transecting these sensitive organs. The postoperative pain of the recipient was controlled with subcutaneous buprenorphine every 6 h, up to 72 h post-surgery [69].

### Tracheostomy

Tracheostomy needs skin and fascia incisions, as well as dissection of the muscles and trachea, which all contribute to somatic pain. Considering the few nociceptors available in the trachea, it seems that visceral pain is not associated with tracheostomy-induced pain.

Tracheostomy is usually a preparation for a main surgical procedure. The choice of anesthetic agent for tracheostomy depends on the requirements of the main surgical model. As reported, intraperitoneal injection of pentobarbital sodium/ketamine [70], urethane/alpha-chloralose [71], and ketamine/xylazine [72], as well as inhaled isoflurane have been applied as anesthetic agents in this surgical procedure. The combination of pentobarbital/Ketamine/xylazine decreases the tracheal mucociliary clearance [73]. However, isoflurane, propofol, or fentanyl/dexmedetomidine/midazolam do not seem to change the tracheal mucociliary clearance values [73]. Opioid analgesics such as buprenorphine should be used as intra-operative analgesics. Due to the carcinogenic effects of urethane, we do not recommend using this agent for anesthesia.

### Laparotomy

The abdominal cavity contains several viscera that are important in biomedical research. Laparotomy is the main approach to access these viscera. Laparotomy pain may originate from the soft tissues in and around the abdominal incision site including the skin, fascia, muscles, and ligaments. The superficial nerves may be compressed following surgery due to inflammation, scar tissue formation, muscle length unfitness, or hyperexcitability of the nerves, and may lead to neuralgia. Manipulation of viscera may cause acute (surgical) and chronic (postsurgical) pain and should be targeted appropriately [74]. For the first 24 h after laparoscopic procedures, it is believed that visceral pain constitutes the major part of overall pain [75, 76].

The most typical anesthetic agents used in mice models of laparotomy include inhaled anesthetics, such as isoflurane and sevoflurane, and injectable anesthetics, like ketamine/xylazine/acepromazine. Intraperitoneal injection of drugs for laparotomy has been a topic of debate. However, various reports have used this method [77, 78]. In general, we suggest avoiding abdominal lavage if the intraperitoneal route is used for anesthesia. Subcutaneous administration of lidocaine (10 mg/kg) with or without bupivacaine (3 mg/kg) at the intended site for the incision (line block) [79], provides multimodal analgesia and decreases the dose of the main anesthetics.

#### Liver and bile duct

For bile duct ligation, isoflurane or sevoflurane [80, 81] are used. In rats, it is shown that isoflurane induces liver injury, mainly due to a regulation of expression of insulin-like growth factor-1 [82]. However, isoflurane has lower hepatotoxicity, in comparison to other halogenated anesthetics (e.g., halothane) [83–85]. In horses, it is shown that anesthesia with isoflurane increases hepatic bilirubin excretion, but has a modest effect on hepatic bilirubin formation or clearance [86]. In humans’ serum samples taken 3–14 days after anesthesia, sevoflurane is shown to less frequently induce elevation of liver enzymes than isoflurane [87].

Besides using inhalation anesthetics, other systemic anesthetic agents such as a mixture of ketamine/xylazine/acepromazine have also been used [40, 88]. It is shown that ketamine/xylazine/acepromazine is a safe and effective protocol for liver transplantation surgery in mice [89]. Ketamine is shown to have hepatoprotective effects [90]. Postoperative pain of the bile duct ligation surgery could be alleviated by buprenorphine [91].

Partial hepatectomy is performed by isoflurane anesthesia along with meloxicam and buprenorphine analgesia. It is shown that flunixin meglumine could not provide enough analgesia in this surgery [92].

#### Kidneys

For renal models, pain may be induced by renal mechanosensory nerves (pressure-responsive) and renal chemosensory nerves (responsive to ischemia or renal interstitial fluid alterations) [93]. This is in addition to the general mechanisms of pain related to laparotomies. The proper anesthetic protocol should preserve renal functions during kidney studies. To induce renal ischemia–reperfusion injury (RIRI), a combination of ketamine/xylazine [94] or ketamine/medetomidine [95] is used. Although ketamine metabolites are eliminated by kidneys, it is generally safe to use it with models of compromised kidneys, such as RIRI. However, it is recommended to reduce the dose of xylazine in renal failure models [7]. Therefore, to provide required analgesia, buprenorphine should be used along with ketamine/xylazine or ketamine/medetomidine anesthesia [95].

For the renal ischemia model, the ketamine/xylazine combination is not recommended due to its short duration of surgical anesthesia (~ 20 min.), which is not adequate for a 1-h procedure. Isoflurane is also not a viable option due to its intrinsic renoprotective activity, which could counteract the effect of the surgical procedure [96]. Intraperitoneal injection of pentobarbital has been used for the renal ischemia model [96]. However, it should be combined with a potent analgesic (e.g., buprenorphine).

Buprenorphine is a recommended analgesia for mouse models of RIRI and ureteral obstruction [94, 97–99]. Since buprenorphine is metabolized by the liver, it can be used in models of compromised kidney functions [100]. Due to the renal side effects of NSAIDs, they should not be used as analgesic agents in RIRI models [58, 94].

For performing the renal micropuncture technique, thiobutabarbital sodium (Inactin®) has been used [101]. However, this short-acting barbiturate derivative does not possess analgesic effects and is not recommended to be used alone. Also, mice’s response to thiobutabarbital is not consistent [102]. Accordingly, ketamine has been used in combination with thiobutabarbital [101, 103]. Furthermore, barbiturates, like sodium pentobarbital are counter-indicated in RIRI models. In fact, they reduce blood pressure that would subsequently reduce kidney blood flow, glomerular filtration rate, and urine excretion [94]. Halothane and isoflurane may have fewer side effects on vascular activities and can be applied [101].

Due to better preservation of the renal function, α-chloralose seems to be an effective anesthetic agent for studying renal function in acute interventions [103]. However, α-chloralose lacks analgesic properties and is usually used for non-survival procedures [6, 104]. Therefore, we recommend considering other anesthetic protocols for this purpose.

For ureteral obstruction development, isoflurane [6, 105, 106] or pentobarbital [107] is used. Intra-operative analgesia is achieved using subcutaneous buprenorphine. Postoperative analgesia is maintained by local infiltration of bupivacaine at the incision site and continuation of buprenorphine injection for two days after surgery [106].

Goldblatt model of hypertension requires surgically induced renal arterial stenosis and may have three subtypes: two-kidney one-clip, two-kidney two-clip, and one-kidney one-clip. For these models, anesthetic agents with a minimum effect on renal or cardiovascular function are preferred. Isoflurane [108], pentobarbital [109], ketamine/xylazine [110–112], or ketamine/xylazine/acepromazine [113] are used for this model. Among these, ketamine/xylazine/acepromazine seems to better provide surgical anesthesia. Buprenorphine is used for post-surgical analgesia in this model [114].

#### Abdominal aorta

For abdominal aortic constriction, isoflurane [115, 116] and ketamine/xylazine [117] are used. Although using a ketamine/xylazine mixture is more frequent than that of isoflurane, this combination is not generally advised for major surgeries in mice [22]. Isoflurane should be used with a potent analgesic (e.g., buprenorphine).

#### Gastrointestinal tract

Gastrointestinal (GI) pain, as a form of visceral pain, may be induced by GI disorders or manipulation. For GI surgeries, anesthesia can be induced and maintained with isoflurane [118, 119] or sevoflurane. Buprenorphine is used along with these inhalational agents to provide analgesia [102, 119]. Isoflurane is shown to reduce GI propulsion in rats [120, 121]. Sevoflurane could change the composition of the intestinal microbiome in mice [122]. Breathing anesthetics are more convenient for both the operator and the animals [6, 102]. However, intraperitoneal injection of a ketamine/xylazine/acepromazine mixture or pentobarbital/buprenorphine is also used. Studies have shown no effect of ketamine on intestinal peristalsis in dogs, while they have shown shortened or prolonged peristalsis in pigs [123]. Ketamine is shown to protect the intestines from ischemic damage [123] and safeguard the stomach from lipopolysaccharide-induced injury [102]. Buprenorphine reduces GI motility and slows the passage of feed in the GI tract [124].

Dutton et al*.* [125] concluded that pentobarbital may induce local inflammation, and therefore it is not recommended for inflammatory bowel disease models. Dexmedetomidine is shown to significantly inhibit intestinal peristalsis in endotoxemic mice [126], suggesting that this drug should be used with caution in ileus models [126].

Buprenorphine is the routine analgesic for GI surgeries. It should be administered 1 h preceding the incision, with a second dose 6–12 h following the first dose. The next doses are prescribed within 6–12 h intervals, up to 72 h post-surgery [22].

#### Testes

Semen collection may be performed from the female reproductive tract after mating. This requires euthanasia of the female animal and resection of the whole reproductive tract. In this regard, an overdose of intraperitoneal thiopental or pentobarbital (200 mg/kg) with concentration of 60 mg/mL or intraperitoneal ketamine/xylazine with euthanasia dose (300–400 mg/kg + 30–40 mg/kg) can be used [127]. Another method for semen collection is through electroejaculation of the male mice, using a rectal probe. The mouse may be anesthetized with intraperitoneal ketamine/xylazine or intraperitoneal pentobarbitone [128]. It is shown that pentobarbitone anesthesia led to higher spermatozoa count and less urine contamination in the collected semen [128]. However, it should be noted that pentobarbitone induces severe respiratory depression in mice [129].

#### Ovaries

Oophorectomy or ovariectomy may be performed using isoflurane [130] or halothane [131, 132]. Intraperitoneal injection of ketamine/medetomidine has also been used for general anesthesia for this experimental procedure [132]. Intra-operative and postoperative analgesia may be achieved by opioids (e.g., buprenorphine) and NSAIDs (e.g., ketoprofen).

For oocyte collection, 2% tribromoethanol intraperitoneally or inhalation of isoflurane is used [133]. In oocyte retrieval surgery, any agent administered intraperitoneally may come in direct contact with the oviduct or cumulus-oocyte mass and could have toxic effects on oocytes. It is shown that mice anesthetized with intraperitoneal tribromoethanol have a higher proportion of dead oocytes, in comparison to mice euthanized just before the oocyte collection or mice anesthetized with isoflurane [133]. Therefore in these surgeries, it is recommended to use inhalation anesthesia, intravenous or intramuscular injectable anesthesia [134].

### Skull

Surgeries involving the skull are categorized as brain surgeries or stroke induction.

#### Brain

Brain surgery is usually performed under stereotaxic frame guidance and involves injury to soft tissues (skin, muscles), and the osseous skull. Isoflurane [135–137] or ketamine/xylazine [136] are used for stereotaxic surgeries. Isoflurane anesthesia is combined with subcutaneous buprenorphine for intra-operative and postoperative analgesia for stereotaxic surgeries [135].

In one study [135], buprenorphine was injected just before making the surgical incision; though to achieve preemptive analgesia, we recommend the administration of buprenorphine 1 h before making the incision. In order to achieve multimodal analgesia, lidocaine (10 mg/kg) with or without bupivacaine (3 mg/kg) could be injected subcutaneously [79], just before making the skin incision. Also, before drilling or cutting the skull, its periosteum should be anesthetized with a drop of 1% lidocaine splash block [137]. It is also recommended to apply another lidocaine splash block to the periosteum after a few drillings [137]. It is shown that subcutaneous buprenorphine provides more effective analgesia, compared to commonly used NSAIDs (such as carprofen and meloxicam), following craniotomy [135]. Also, buprenorphine reduces the pain and distress in the subarachnoid hemorrhage model, but meloxicam or carprofen is unable to achieve this [138]. However, concurrent use of carprofen or meloxicam with buprenorphine reduces tissue inflammation after surgery. Subcutaneous dexamethasone (0.2 mg/kg) has been used to prevent brain swelling in craniotomy models [137, 139]. Carprofen is used for suppressing inflammation and immune response in a chronic model of the cranial window for cortical activity imaging [140].

For inducing seizures, stereotaxic intrahippocampal administration of kainic acid is performed under isoflurane anesthesia, combined with buprenorphine. Since isoflurane has no effect on ion channel function, this agent is recommended for the kainic acid-induced seizure model [136]. Ketamine/xylazine along with local anesthesia may also be used for this purpose, though ketamine may delay the onset of seizures due to its blocking effects on the NMDA channels [136].

#### Stroke

Ketamine and barbiturates are known to have a neuroprotective effect and may interfere with the effect of the test substance in stroke models. Due to this side effect, inhalation anesthesia is preferred for the induction of experimental stroke models [141]. Mechanical ventilation during isoflurane anesthesia seems to reduce the undesirable effects of anesthesia on the stroke model [141]. In a middle cerebral artery occlusion model, it was shown that postoperative meloxicam significantly reduced and confounded the stroke volume. Buprenorphine did not show this effect and was declared to be the proper analgesia for this stroke model [142, 143].

### Spinal cord

Laminectomy is orthopedic surgery. Pain from spinal cord injury (SCI) is a type of neuropathic pain that is categorized as chronic pain [144]. Isoflurane has been used for developing the SCI model. Buprenorphine is used as postoperative analgesia for 2–3 days and after that, as needed per animal [145, 146]. In rats, buprenorphine could be used as an analgesic following SCI models, without behavioral, physiological, or anatomical effects [147]. It is shown that buprenorphine is more potent than morphine in alleviating neuropathic pain caused by SCI [147, 148].

Intraperitoneal injection of ketamine/xylazine is used to transplant neural stem cells into the spinal cord [149]. Chloral hydrate is an agent frequently used in studies of SCI or spared nerve injury [150, 151]. However, this agent is classified as a sedative without analgesic properties, and can only be used as a pre- or postoperative medication [152]. Furthermore, it is now considered an antiquated agent with serious animal welfare concerns [6]. Therefore, it should be avoided and replaced with other agents.

Tribromoethanol may rarely be used as an anesthetic in rodents [153]. This compound is very unstable and has specific storage conditions. Following intraperitoneal injection, it may cause severe inflammation and peritonitis in animals [154]. Due to the adverse effects of tribromoethanol, it is recommended to avoid using this agent [22].

Ketamine/xylazine are used despite the fact that they provide short-term surgical anesthesia (~ 20 min.), while laminectomy is a longer procedure. Therefore, we do not recommend this compound. To make a model of complete-crush transection SCI, mice were anesthetized using isoflurane [155]. However, this protocol lacks the analgesic component and we suggest a combined use of buprenorphine injection along with local analgesia at the site of laminectomy and spinal cord crushing.

It is shown that microinjection of lidocaine through a lumbar puncture following SCI in rats, prevents adverse cellular events related to the injury, and promotes behavioral recovery of the animal [156].

### Thoracotomy

Thoracotomy may involve both somatic and visceral pains. When the intercostal approach is used, the pain has resulted from skin incision, dissection of the intercostal muscles that are heavily innervated, and manipulation of the thoracic visceral organs. When the thoracotomy involves hard tissues as in sternotomy or costotomy, disrupting the hard tissues of the sternum or ribs add to the overall pain of the procedure.

In many thoracotomy procedures, using anesthetic protocols with minimum cardiovascular side effects is of utmost importance. In this regard, urethane, sodium pentobarbital, and ketamine/xylazine have been shown to induce substantial depression in the cardiovascular system and are best to avoid when the (patho)physiology of the system is in question. It is suggested that isoflurane has minimum cardiovascular side effects and has a rapid recovery [157]. This is also shown that isoflurane has fewer effects on hemodynamics than ketamine/xylazine, urethane, or pentobarbital sodium [25].

Although anesthetic agents such as pentobarbital sodium [158], isoflurane [159], or ketamine/xylazine [160, 161] have been extensively used in literature, thoracotomy is considered one of the most painful procedures, and the major drawbacks of these agents are their poor, if any, analgesic effect [22, 162, 163].

To ensure a humane procedure, a potent analgesic regimen should be applied. In one study, anesthesia is induced by an intraperitoneal injection of a combination of midazolam/medetomidine/fentanyl. Afterward, the anesthesia is maintained by isoflurane and mechanical ventilation [164]. As an alternative, a mixture of ketamine and medetomidine may be used [164], in which we suggest adding buprenorphine 1 h before the first surgical incision.

In another study, anesthesia was induced in an anesthetic chamber containing isoflurane [165]. Afterward, intraperitoneal injection of a mixture of buprenorphine (0.1 mg/kg) and etomidate (10 mg/kg) was given. Anesthesia was continued initially by administrating 2% isoflurane with an oxygen flow rate of 80 ml/min [165], which was later reduced to 1–1.5% isoflurane to achieve a proper depth of anesthesia [166]. To connect the animal to the anesthetic system, endotracheal intubation was performed using a 3-cm shortened plastic cannula (G20). The tube was connected to a rodent ventilator with set values for the ventilation rate = 53.5 × M^−0.26^, and tidal volume = 6.2 × M^1.01^; in which M is the bodyweight of the animal in grams [165, 166]. Tidal volume was set to obtain a peak inspiratory pressure of 11 ± 1 cmH_2_O [165]. To avoid the interruption of normal respiration with ventilations, pancuronium can be injected intraperitoneally. Using neuromuscular blocking agents (e.g., pancuronium) complicates anesthetic monitoring. Therefore, at least continuous monitoring of the heart rate should be used as an indication of the depth of anesthesia [7, 165]. Positive end-expiratory pressure can be established before incising the diaphragm by placing the tip of the expiratory ventilation tube, 2 cm below the surface of the water [165]. For postoperative analgesia, buprenorphine is suggested for at least three days post-operatively [164]. However, other references recommend using pure opioid agonists (e.g., morphine, fentanyl) for at least the first 48 h post-surgery. Respiratory rate and quality should be monitored to avoid opioid-related respiratory depression. Systemic opioid therapy should be continued with partial agonists (e.g., buprenorphine) [167]. NSAIDs such as meloxicam, ketoprofen, or carprofen should also be used. Intercostal nerve block (e.g., lidocaine and/or bupivacaine) injection at the intercostal incision site and two intercostal spaces, cranial and caudal to the incision site, may also be used before closing the incision. This may be used in addition to opioids [58, 168]. Signs of pain should be monitored for at least 5 days after surgery.

### Sepsis

Three main types of mouse models of sepsis are 1) administration of toxins of bacteria, 2) injection of an exact amount of a purified live bacterium or fecal-derived bacteria (polymicrobial infection), and 3) surgical ligation and puncture/destruction of the intestinal tract (mainly ileum-cecum portion). These models, especially the surgical type, can induce a high level of pain for the animals and require proper analgesia and anesthesia [169–172].

For surgical modeling, inhalational anesthesia with isoflurane is recommended. A combination of ketamine/xylazine is also suggested for anesthesia followed by at least two days of buprenorphine for postoperative analgesia [173].

Although analgesia is required for septic models [172], there are concerns related to the increased death rate due to the use of analgesics in these models. This has led to low consumption of analgesics in septic model studies. In fact, only 15% of studies that used the septic model reported using analgesics [174, 175]. This concern may be truly related to NSAIDs and morphine that can elevate the level of circulating IL-6. However, other opioid analgesics such as buprenorphine have low side effects and immunosuppressive activity and should be used to control the pain [174, 176, 177]. Buprenorphine can be used subcutaneously, immediately before induction of the model, and then every 12 h for at least two days [169, 174]. Besides, it is recommended to use 50–100 μL of lidocaine or bupivacaine (0.25%) in the surgical incision site to reduce the dosage of buprenorphine by half, for the first injection [177].

#### Wound models

Wound models include incisional or excisional cutaneous wounds and cause somatic pain. Ketamine/xylazine is used for excisional [178, 179] and incisional wound models. In rats, it is shown that ketamine/xylazine and thiopental could alter the expression of several genes related to wound healing [180].

Medetomidine/midazolam/butorphanol (0.3 mg/kg, 4 mg/kg, and 5 mg/kg) is shown to provide surgical anesthesia identical to ketamine/xylazine anesthesia [181]. Isoflurane is used for both types of wound models [182, 183]. Pentobarbital is suggested for excisional wounds [184], though it does not provide analgesia and should be accompanied by a local analgesic, such as bupivacaine or lidocaine [185].

Postoperative analgesia for wound healing research could be provided by short-term use of systemic or topical opioids, short-term use of NSAIDs (e.g., carprofen or meloxicam), immediate use of local anesthetics at the time of wound creation, or tramadol local infiltration at the injury site [185]. These modalities have not shown any adverse effect on wound healing [185]. Other analgesics, such as acetaminophen, gabapentin, or ketamine may also be considered adjunct therapies for wound pain management [185].

## Conclusions

In conclusion, a proper method of surgical anesthesia includes both the anesthetic and analgesic components. Furthermore, post-surgical analgesia should be provided for almost all surgical procedures. Methods of anesthesia and analgesia for the below procedures on mice were presented in this paper: injections and catheterizations, working with agitated animals, temporary and long-term immobilization for non-painful procedures, imaging, ophthalmic procedures, limbs procedures, tracheostomy, laparotomy (liver, bile duct, kidneys, abdominal aorta, gastrointestinal tract, testes, and ovaries), skull (brain and stroke), spinal cord, thoracotomy, sepsis, and wound models. We saw that the induction and maintenance of anesthesia in mice could be achieved concurrently. In this regard, we suggested various injectable and inhalational drugs. Although ketamine and xylazine are common drugs in mice anesthesia, their use should be limited to minor surgeries. For major surgeries one should consider the addition of acepromazine to this combination or use potent analgesics. Isoflurane is a common inhalational drug in mice. It could be used as an open-drop method for non-surgical procedures or be accurately administered using a vaporizer and ventilator. We also critically examined some of the current literature according to the quality of their anesthesia/analgesia modalities. We found that many improper modalities lack the analgesic component and only provided general anesthesia, but not surgical anesthesia. Overall, this paper provides supported discussions on the choose of anesthesia/analgesia methods according to the type of the intended procedures.

## Supplementary Information

**Additional file 1.** Anesthetic drugs used for laboratory mice. The doses of common anesthetic drugs used for laboratory mice are presented in this supplement.

**Additional file 2.** Analgesic drugs for laboratory mouse. The doses of common analgesic drugs used for laboratory mice are presented in this supplement.

**Additional file 3.** Pharmacology of common anesthetic drugs in adult mice. The pharmacology of common anesthetic drugs in adult mice are discussed in this supplement.

## Data Availability

All relevant data are presented in the article.

## Abbreviations

A&A: Anesthesia and analgesia
FDG: Blood fluorodeoxyglucose
GI: Gastrointestinal
IM: Intramuscular
IOP: Intraocular pressure
NSAIDs: Nonsteroidal anti-inflammatory drugs
PET: Positron emission tomography
RIRI: Renal ischemia-reperfusion injury
SCI: Spinal cord injury
