# Supplementary material for: Anesthesia and analgesia for common research models of adult mice
Source: Lab Anim Res. 2022 Dec 13;38:40. doi: 10.1186/s42826-022-00150-3 (PMC9746144; doi:10.1186/s42826-022-00150-3)
Supplement: Supplementary file 1 — Additional file 1. Anesthetic drugs used for laboratory mice. The doses of common anesthetic drugs used for laboratory mice are presented in this supplement. [file 42826_2022_150_MOESM1_ESM.docx]

**Anesthetic and perianesthetic drugs for laboratory mice. Please refer to the text for information on their indication of use.**

| **Agent** | **Level of analgesia** | **Level of analgesia references** | **Dose** | **Route of Administration** | **Dose references** |
| --- | --- | --- | --- | --- | --- |
| **Inhalational anesthetics** | | | | | |
| Halothane (induction) | No analgesia (should be used in combination with analgesics; see Supplement 2) | ^1^ | 3-4% + oxygen | Inhalational | ^2^ |
| Halothane (maintenance) | No analgesia (should be used in combination with analgesics; see Supplement 2) |  | 1-2% + oxygen | Inhalational | ^2^ |
| Isoflurane (induction) | Analgesia not sufficient for painless surgery (should be used in combination with analgesics; see Supplement 2) | Although some references have shown analgesic properties for isoflurane ^3,4^, this drug is generally not considered as an analgesic and thus cannot be used alone in a balanced anesthesia technique ^5–7^. | 4-5% + oxygen (2 L/min) | Inhalational | ^8,9^ |
|  |  |  | 3.5-4.5% + oxygen (2 L/min) | Inhalational | ^2,10–13^ |
| Isoflurane (maintenance) | Analgesia not sufficient for painless surgery (should be used in combination with analgesics; see Supplement 2) |  | 1.5-3% + oxygen (2 L/min) | Inhalational | ^2,14,15^ |
|  |  |  | 2% + oxygen (2 L/min) in the beginning and then reduced to 1-1.5% + oxygen (2 L/min) to maintain a proper anesthetic depth | Inhalational | ^11,12^ |
|  |  |  | 0.8-1.8% + oxygen (2 L/min) | Inhalational | ^13^ |
| Isoflurane + nitrous oxide | Poor analgesia (should be used in combination with analgesics; see Supplement 2) | ^1^ | 1.0 to 1.5% isoflurane + 60% nitrous oxide + ~ 39% oxygen | Inhalational | ^16^ |
| Sevoflurane | No analgesia (should be used in combination with analgesics; see Supplement 2) | ^1,17^ | 6% + oxygen (0.8–1 L/min) | Inhalational | ^18^ |
| **Injectable anesthetics** | | | | | |
| α-chloralose + ketamine | Poor analgesia (should be used in combination with analgesics; see Supplement 2) | ^5^ | 120 (mg/kg) + 100 (mg/kg) | IP | ^19^ |
| Ketamine + medetomidine | Variable anesthesia/analgesia that is only suitable for minor procedures such as retro-orbital bleeding; (For other procedures should be used in combination with analgesics; see Supplement 2) | ^5,20^ | 75 (mg/kg) + 1 (mg/kg) | IP | ^2,21^ |
|  |  |  | 100–150 (mg/kg)+ 0.25 (mg/kg) | IP | ^22^ |
| Ketamine + xylazine + acepromazine | Analgesia sufficient for laparotomy. For more painful procedures (e.g., thoracotomy, orthopedics, neural surgeries) should be used in combination with additional analgesics (see Supplement 2) | ^7^ | 100 (mg/kg)+ 20 (mg/kg)+ 3 (mg/kg) | IP | ^23^ |
|  |  |  | 100 (mg/kg)+ 10 (mg/kg)+ 3 (mg/kg) | IP | ^24^ |
|  |  |  | 65 (mg/kg)+ 13 (mg/kg)+ 2 (mg/kg; older mice may have more abdominal adipose tissues and require 20% higher doses of anesthetics) | IP | ^25^ |
| Ketamine + xylazine | Sufficient analgesia for minor surgeries involving skin or muscles. For more painful procedures (e.g., laparotomy, craniotomy, thoracotomy, etc.) it should be used in combination with analgesics (see Supplement 2) | ^7^ | 128 (mg/kg)+ 8.5 (mg/kg) | IP | ^26^ |
|  |  |  | 100 (mg/kg)+ 5 (mg/kg) | IP | ^27,28^ |
|  |  |  | 80 (mg/kg)+ 10 (mg/kg) | IP | ^29–31^ |
|  |  |  | 125 (mg/kg)+ 10 (mg/kg) | IP | ^32^ |
|  |  |  | 80 (mg/kg)+ 16 (mg/kg) | IP | ^33^ |
|  |  |  | 100 (mg/kg)+ 10 (mg/kg) | IP | ^34,35^ |
|  |  |  | 100 (mg/kg)+ 8 (mg/kg) | IP | ^36^ |
| Midazolam + medetomidine | Poor analgesia (should be used in combination with analgesics; see Supplement 2) | ^37^ | 5 (mg/kg)+ 0.5 (mg/kg) | IP | ^22,35^ |
| Pentobarbital | No analgesia (should be used in combination with analgesics; see Supplement 2) | ^7^ | 40–50 (mg/kg) | IP | ^23,36,38,39^ |
|  |  |  | 50-60 (mg/kg) | IP | ^40^ |
|  |  |  | 65 (mg/kg) | IP | ^41^ |
|  |  |  | 0.8 mg/mouse | IP | ^42^ |
|  |  |  | 30 (mg/kg) | IP | ^43^. |
| Pentobarbital + ketamine | Poor analgesia (should be used in combination with analgesics; see Supplement 2) | ^5,7^ | 50 (mg/kg)+ 50 (mg/kg) | IP | ^44^ |
| Propofol | Poor analgesia; may be used for induction of anesthesia | ^5,7^ | 26 (mg/kg) | IV | ^6^ |
| Thiobutabarbital (Inactin®) | Inconsistent analgesia (should be used in combination with analgesics; see Supplement 2) | ^7^ | 100 (mg/kg) | IP | ^19^ |
| **Neuromuscular blocking agent** | | | | | |
| Pancuronium | No analgesic or anesthetic effects (must be used in combination with anesthetic and analgesic drugs) | - | 1 (mg/kg) | IP | ^45^ |
| **Anticholinergic** | | | | | |
| Atropine | No analgesic or anesthetic effects | - | 0.05 (mg/kg) | IP | ^46^ |
| **Antidotes** | | | | | |
| Atipamezole (antidote to xylazine, medetomidine, detomidine, dexmedetomidin) | Reverse the analgesic properties of the main drug | - | 1 (mg/kg) | IP | ^2,21^ |
|  |  |  |  |  |  |
| Naloxone (antidote to opioids) | Reverses the analgesic effects of opioids | - | 20 (?) | IP | ^6^ |
|  |  |  | 0.01-0.1 (mg/kg) | IP, IV | ^7^ |
|  |  |  | 0.05-0.1 (mg/kg) | IP, IV | ^47^ |

IP: intraperitoneal; IV: intravenous; PO: oral; SC: subcutaneous.

**References:**

1. Miller AL, Theodore D, Widrich J. *Inhalational Anesthetic*. StatPearls Publishing; 2022. Accessed October 1, 2022. https://www.ncbi.nlm.nih.gov/books/NBK554540/

2. Sophocleous A, Idris AI. Ovariectomy/Orchiectomy in Rodents. In: Idris AI, ed. *Bone Research Protocols*. Vol 1914. Methods in Molecular Biology. Springer New York; 2019:261-267. doi:10.1007/978-1-4939-8997-3_13

3. Kingery WS, Agashe GS, Guo TZ, et al. Isoflurane and nociception: spinal alpha2A adrenoceptors mediate antinociception while supraspinal alpha1 adrenoceptors mediate pronociception. *Anesthesiology*. 2002;96(2):367-374. doi:10.1097/00000542-200202000-00023

4. Maud P, Thavarak O, Cédrick L, et al. Evidence for the use of isoflurane as a replacement for chloral hydrate anesthesia in experimental stroke: an ethical issue. *BioMed Res Int*. 2014;2014:802539. doi:10.1155/2014/802539

5. Grimm KA, Lamont LA, Tranquilli WJ, Greene SA, Robertson SA. *Veterinary Anesthesia and Analgesia*. Fifth edition. Wiley Blackwell; 2015.

6. Fish R, Danneman PJ, Brown M, Karas A. *Anesthesia and Analgesia in Laboratory Animals*. Academic Press; 2011.

7. Flecknell PA. *Laboratory Animal Anaesthesia*. Fourth edition. Elsevier/AP, Academic Press is an imprint of Elsevier; 2016.

8. Tag CG, Weiskirchen S, Hittatiya K, Tacke F, Tolba RH, Weiskirchen R. Induction of experimental obstructive cholestasis in mice. *Lab Anim*. 2015;49(1_suppl):70-80.

9. Van Campenhout S, Van Vlierberghe H, Devisscher L. Common bile duct ligation as model for secondary biliary cirrhosis. In: *Experimental Cholestasis Research*. Springer; 2019:237-247.

10. Souza VR, Mendes E, Casaro M, Antiorio ATFB, Oliveira FA, Ferreira CM. Description of Ovariectomy Protocol in Mice. In: Guest PC, ed. *Pre-Clinical Models*. Vol 1916. Methods in Molecular Biology. Springer New York; 2019:303-309. doi:10.1007/978-1-4939-8994-2_29

11. Pacher P, Nagayama T, Mukhopadhyay P, Bátkai S, Kass DA. Measurement of cardiac function using pressure–volume conductance catheter technique in mice and rats. *Nat Protoc*. 2008;3(9):1422-1434. doi:10.1038/nprot.2008.138

12. Nicks AM, Kesteven SH, Li M, et al. Pressure overload by suprarenal aortic constriction in mice leads to left ventricular hypertrophy without c-Kit expression in cardiomyocytes. *Sci Rep*. 2020;10(1):15318. doi:10.1038/s41598-020-72273-3

13. Drysch M, Wallner C, Schmidt SV, et al. An optimized low-pressure tourniquet murine hind limb ischemia reperfusion model: Inducing acute ischemia reperfusion injury in C57BL/6 wild type mice. *PLOS ONE*. 2019;14(1):e0210961. doi:10.1371/journal.pone.0210961

14. Li C, Zhang YY, Frieler RA, et al. Myeloid Mineralocorticoid Receptor Deficiency Inhibits Aortic Constriction-Induced Cardiac Hypertrophy in Mice. *PLoS ONE*. 2014;9(10). doi:10.1371/journal.pone.0110950

15. Byers SL, Wiles MV, Taft RA. Surgical Oocyte Retrieval (SOR): a Method for Collecting Mature Mouse Oocytes Without Euthanasia. *J Am Assoc Lab Anim Sci*. 2009;48(1):8.

16. Goto T, Fukuyama N, Aki A, et al. Search for appropriate experimental methods to create stable hind-limb ischemia in mouse. *Tokai J Exp Clin Med*. 2006;31(3):128-132.

17. Grover S, Wilkinson DJ. Sevoflurane and analgesia. *BJA Br J Anaesth*. 2007;98(5):691-692. doi:10.1093/bja/aem077

18. Van Campenhout S, Van Vlierberghe H, Devisscher L. Common Bile Duct Ligation as Model for Secondary Biliary Cirrhosis. *Methods Mol Biol Clifton NJ*. 2019;1981:237-247. doi:10.1007/978-1-4939-9420-5_15

19. Rieg T, Richter K, Osswald H, Vallon V. Kidney function in mice: thiobutabarbital versus ?-chloralose anesthesia. *Naunyn Schmiedebergs Arch Pharmacol*. 2004;370(4):320-323. doi:10.1007/s00210-004-0982-x

20. Fish RE, Brown MJ, Danneman PJ, Karas AZ. *Anesthesia and Analgesia in Laboratory Animals*. 2nd ed. Academic Press; 2008.

21. Aerts J, Nys J, Arckens L. A Highly Reproducible and Straightforward Method to Perform In Vivo Ocular Enucleation in the Mouse after Eye Opening. *J Vis Exp JoVE*. 2014;(92):51936. doi:10.3791/51936

22. Borst O, Ochmann C, Schönberger T, et al. Methods Employed for Induction and Analysis of Experimental Myocardial Infarction in Mice. *Cell Physiol Biochem*. 2011;28(1):1-12. doi:10.1159/000331708

23. Arras M, Autenried P, Rettich A, Spaeni D, Rülicke T. Optimization of Intraperitoneal Injection Anesthesia in Mice: Drugs, Dosages, Adverse Effects, and Anesthesia Depth. *Comp Med*. 2001;51(5):14.

24. Buitrago S, Martin TE, Tetens-Woodring J, Belicha-Villanueva A, Wilding GE. Safety and efficacy of various combinations of injectable anesthetics in BALB/c mice. *J Am Assoc Lab Anim Sci JAALAS*. 2008;47(1):11-17.

25. Mason JB, Parkinson KC, Habermehl TL. Orthotopic Ovarian Transplantation Procedures to Investigate the Life- and Health-span Influence of Ovarian Senescence in Female Mice. *J Vis Exp JoVE*. 2018;(132):56638. doi:10.3791/56638

26. Hoffman-Goetz L, Quadrilatero J, Boudreau J, Guan J. Adrenalectomy in mice does not prevent loss of intestinal lymphocytes after exercise. *J Appl Physiol Bethesda Md 1985*. 2004;96(6):2073-2081. doi:10.1152/japplphysiol.01262.2003

27. Fujino T, Nakagawa N, Yuhki K ichi, et al. Decreased susceptibility to renovascular hypertension in mice lacking the prostaglandin I2 receptor IP. *J Clin Invest*. 2004;114(6):805-812. doi:10.1172/JCI200421382

28. Brenes RA, Jadlowiec CC, Bear M, et al. Toward a mouse model of hind limb ischemia to test therapeutic angiogenesis. *J Vasc Surg*. 2012;56(6):1669-1679. doi:10.1016/j.jvs.2012.04.067

29. Gage GJ, Kipke DR, Shain W. Whole Animal Perfusion Fixation for Rodents. *J Vis Exp*. 2012;(65):3564. doi:10.3791/3564

30. Mirabelli E. Pathological pain processing in mouse models of multiple sclerosis and spinal cord injury: contribution of plasma membrane calcium ATPase 2 (PMCA2). Published online 2019:19.

31. Lee Jason J., Arpino John-Michael, Yin Hao, et al. Systematic Interrogation of Angiogenesis in the Ischemic Mouse Hind Limb. *Arterioscler Thromb Vasc Biol*. 2020;40(10):2454-2467. doi:10.1161/ATVBAHA.120.315028

32. Vandivort TC, An D, Parks WC. An improved method for rapid intubation of the trachea in mice. *JoVE J Vis Exp*. 2016;(108):e53771.

33. Le Clef N, Verhulst A, D’Haese PC, Vervaet BA. Unilateral Renal Ischemia-Reperfusion as a Robust Model for Acute to Chronic Kidney Injury in Mice. Chatziantoniou C, ed. *PLOS ONE*. 2016;11(3):e0152153. doi:10.1371/journal.pone.0152153

34. Carbajal KS, Weinger JG, Whitman LM, Schaumburg CS, Lane TE. Surgical Transplantation of Mouse Neural Stem Cells into the Spinal Cords of Mice Infected with Neurotropic Mouse Hepatitis Virus. *JoVE J Vis Exp*. 2011;(53):e2834. doi:10.3791/2834

35. Aref Z, de Vries MR, Quax PHA. Variations in Surgical Procedures for Inducing Hind Limb Ischemia in Mice and the Impact of These Variations on Neovascularization Assessment. *Int J Mol Sci*. 2019;20(15). doi:10.3390/ijms20153704

36. Tecirlioglu RT, Hayes ES, Trounson AO. Semen collection from mice: electroejaculation. *Reprod Fertil Dev*. 2002;14(6):363. doi:10.1071/RD02015

37. Kirihara Y, Takechi M, Kurosaki K, Kobayashi Y, Kurosawa T. Anesthetic effects of a mixture of medetomidine, midazolam and butorphanol in two strains of mice. *Exp Anim*. 2013;62(3):173-180. doi:10.1538/expanim.62.173

38. Hildebrandt IJ, Su H, Weber WA. Anesthesia and Other Considerations for in Vivo Imaging of Small Animals. *ILAR J*. 2008;49(1):17-26. doi:10.1093/ilar.49.1.17

39. Johns C, Gavras I, Handy DE, Salomao A, Gavras H. Models of experimental hypertension in mice. *Hypertens Dallas Tex 1979*. 1996;28(6):1064-1069. doi:10.1161/01.hyp.28.6.1064

40. Wei Q, Dong Z. Mouse model of ischemic acute kidney injury: technical notes and tricks. *Am J Physiol-Ren Physiol*. 2012;303(11):F1487-F1494. doi:10.1152/ajprenal.00352.2012

41. Giovanoli S, Notter T, Richetto J, et al. Late prenatal immune activation causes hippocampal deficits in the absence of persistent inflammation across aging. *J Neuroinflammation*. 2015;12(1):221. doi:10.1186/s12974-015-0437-y

42. Tsuchiya Y, Sawada S, Yoshioka I, et al. Increased surgical stress promotes tumor metastasis. *Surgery*. 2003;133(5):547-555. doi:10.1067/msy.2003.141

43. Oyama A, Funayama E, Hayashi T, Saito A, Yamamoto Y. A New Model of Acquired Lymphedema in the Mouse Hind Limb: A Preliminary Report. *Ann Plast Surg*. 2012;69(5):565-568. doi:10.1097/SAP.0b013e31821ee3dd

44. Hoffmeyer MR, Scalia R, Ross CR, Jones SP, Lefer DJ. PR-39, a potent neutrophil inhibitor, attenuates myocardial ischemia-reperfusion injury in mice. *Am J Physiol Heart Circ Physiol*. 2000;279(6):H2824-2828. doi:10.1152/ajpheart.2000.279.6.H2824

45. Bacmeister L, Segin S, Medert R, Lindner D, Freichel M, Camacho Londoño JE. Assessment of PEEP-Ventilation and the Time Point of Parallel-Conductance Determination for Pressure-Volume Analysis Under β-Adrenergic Stimulation in Mice. *Front Cardiovasc Med*. 2019;6. doi:10.3389/fcvm.2019.00036

46. Jamal MA, Ahmed AM, Tahir M, et al. Safety and efficacy of ketamine xylazine along with atropine anesthesia in BALB/c mice. *Braz J Pharm Sci*. 2019;55:e17231. doi:10.1590/s2175-97902019000317231

47. Hawk CT, Leary SL, Morris TH, American College of Laboratory Animal Medicine, European College of Laboratory Animal Medicine. *Formulary for Laboratory Animals*. 3rd ed. Blackwell Pub.; 2005.
