# Supplementary material for: Anesthesia and analgesia for common research models of adult mice
Source: Lab Anim Res. 2022 Dec 13;38:40. doi: 10.1186/s42826-022-00150-3 (PMC9746144; doi:10.1186/s42826-022-00150-3)
Supplement: Supplementary file 2 — Additional file 2. Analgesic drugs for laboratory mouse. The doses of common analgesic drugs used for laboratory mice are presented in this supplement. [file 42826_2022_150_MOESM2_ESM.docx]

**Analgesic drugs for laboratory mouse. Please see the main text for more information on the indications and usage of each drug.**

| **Agent** | **Dose** | **Route of Administration** | **Reference** |
| --- | --- | --- | --- |
| **Opioids** | | | |
| Buprenorphine | 0.03 - 0.1 (mg/kg) | SC, IP | ^1–11^ |
| Buprenorphine (sustained-release) | 1 (mg/kg) | SC | ^12^ |
| Butorphanol | 5 (mg/kg) | SC | ^13^ |
| Fentanyl | 0.05 (mg/kg) | IP | ^9^ |
|  | 0.025-0.6 (mg/kg) | SC | ^13^ |
| Meperidine | 20 (mg/kg) | IP | ^13^ |
| Morphine | 10 (mg/kg) | SC | ^13^ |
| Tramadol | 20-40 (mg/kg) | IP | ^13^ |
| **Non-steroidal anti-inflammatory drugs** | | | |
| Carprofen | 5 (mg/kg) | SC | ^14,15^ |
| Diclofenac | 9-28 (mg/kg) | IP | ^13^ |
| Flunixin meglumine | 4-11 (mg/kg) | IV | ^13^ |
| Ibuprofen | 40 (mg/kg) | PO | ^13^ |
| Meloxicam | 5 (mg/kg) | SC | ^13^ |
|  | 1 (mg/kg) | IP | ^16^ |
| **Local analgesics** | | | |
| Bupivacaine | 3 (mg/kg) | local infiltration | ^17^ |
| Lidocaine | 10 (mg/kg) | local infiltration | ^17^ |
| **Others** | | | |
| Acetaminophen | 110-305 (mg/kg) | PO | ^13^ |

IP: intraperitoneal; IV: intravenous; PO: oral; SC: subcutaneous.

**References:**

1. Tag CG, Weiskirchen S, Hittatiya K, Tacke F, Tolba RH, Weiskirchen R. Induction of experimental obstructive cholestasis in mice. *Lab Anim*. 2015;49(1 Suppl):70-80. doi:10.1177/0023677214567748

2. Van Campenhout S, Van Vlierberghe H, Devisscher L. Common Bile Duct Ligation as Model for Secondary Biliary Cirrhosis. *Methods Mol Biol Clifton NJ*. 2019;1981:237-247. doi:10.1007/978-1-4939-9420-5_15

3. Hoffman-Goetz L, Quadrilatero J, Boudreau J, Guan J. Adrenalectomy in mice does not prevent loss of intestinal lymphocytes after exercise. *J Appl Physiol Bethesda Md 1985*. 2004;96(6):2073-2081. doi:10.1152/japplphysiol.01262.2003

4. Wei Q, Dong Z. Mouse model of ischemic acute kidney injury: technical notes and tricks. *Am J Physiol-Ren Physiol*. 2012;303(11):F1487-F1494. doi:10.1152/ajprenal.00352.2012

5. Nicks AM, Kesteven SH, Li M, et al. Pressure overload by suprarenal aortic constriction in mice leads to left ventricular hypertrophy without c-Kit expression in cardiomyocytes. *Sci Rep*. 2020;10(1):15318. doi:10.1038/s41598-020-72273-3

6. Lilley E, Armstrong R, Clark N, et al. Refinement of Animal Models of Sepsis and Septic Shock. *Shock*. 2015;43(4):304-316. doi:10.1097/SHK.0000000000000318

7. Carpenter KC, Hakenjos JM, Fry CD, Nemzek JA. The influence of pain and analgesia in rodent models of sepsis. *Comp Med*. 2019;69(6):546-554.

8. Carbajal KS, Weinger JG, Whitman LM, Schaumburg CS, Lane TE. Surgical Transplantation of Mouse Neural Stem Cells into the Spinal Cords of Mice Infected with Neurotropic Mouse Hepatitis Virus. *JoVE J Vis Exp*. 2011;(53):e2834. doi:10.3791/2834

9. Borst O, Ochmann C, Schönberger T, et al. Methods Employed for Induction and Analysis of Experimental Myocardial Infarction in Mice. *Cell Physiol Biochem*. 2011;28(1):1-12. doi:10.1159/000331708

10. Drysch M, Wallner C, Schmidt SV, et al. An optimized low-pressure tourniquet murine hind limb ischemia reperfusion model: Inducing acute ischemia reperfusion injury in C57BL/6 wild type mice. *PLOS ONE*. 2019;14(1):e0210961. doi:10.1371/journal.pone.0210961

11. Rinkevich Y, Montoro DT, Muhonen E, et al. Denervation of Mouse Lower Hind Limb by Sciatic and Femoral Nerve Transection. *Bio-Protoc*. 2016;6(13):e1865-e1865.

12. Parikh PP, Castilla D, Lassance-Soares RM, et al. A Reliable Mouse Model of Hind limb Gangrene. *Ann Vasc Surg*. 2018;48:222-232. doi:10.1016/j.avsg.2017.10.008

13. Fish R, Danneman PJ, Brown M, Karas A. *Anesthesia and Analgesia in Laboratory Animals*. Academic Press; 2011.

14. Byers SL, Wiles MV, Taft RA. Surgical Oocyte Retrieval (SOR): a Method for Collecting Mature Mouse Oocytes Without Euthanasia. *J Am Assoc Lab Anim Sci*. 2009;48(1):8.

15. Hao Z, Zhao Z, Berthoud HR, Ye J. Development and Verification of a Mouse Model for Roux-en-Y Gastric Bypass Surgery with a Small Gastric Pouch. *PLoS ONE*. 2013;8(1):e52922. doi:10.1371/journal.pone.0052922

16. Aerts J, Nys J, Arckens L. A Highly Reproducible and Straightforward Method to Perform In Vivo Ocular Enucleation in the Mouse after Eye Opening. *J Vis Exp JoVE*. 2014;(92):51936. doi:10.3791/51936

17. Durst MS, Arras M, Palme R, Talbot SR, Jirkof P. Lidocaine and bupivacaine as part of multimodal pain management in a C57BL/6J laparotomy mouse model. *Sci Rep*. 2021;11(1):10918. doi:10.1038/s41598-021-90331-2
