# Supplementary material for: Anesthesia and analgesia for common research models of adult mice
Source: Lab Anim Res. 2022 Dec 13;38:40. doi: 10.1186/s42826-022-00150-3 (PMC9746144; doi:10.1186/s42826-022-00150-3)
Supplement: Supplementary file 3 — Additional file 3. Pharmacology of common anesthetic drugs in adult mice. The pharmacology of common anesthetic drugs in adult mice are discussed in this supplement. [file 42826_2022_150_MOESM3_ESM.docx]

# Pharmacology of common anesthetic drugs in adult mice

| Acepromazine maleate ^1–7^ | |
| --- | --- |
| Classification | CNS agent, Psychotherapeutic, Antipsychotic, Phenothiazine |
| Mechanism of action | Mostly: Post synaptic D_2_ blocker, α_1_ blocker  Less: H_1_ blocker, muscarinic receptor blocker |
| Indications | Tranquilization, Sedation, Pre-anesthesia, Anesthesia (Used alone* or in combination with analgesics or anesthetics such as ketamine, xylazine, isoflurane), Anti-emesis, Anti spasmodic |
| Trade names | Aceproject, Aceprolabs, Atravet, PromAce, Acprozine, Notensil, Plegicil |
| Pharmacokinetic | Metabolism by liver, elimination by urine |
| Dosage forms | Tab: 5, 10, 25 mg, Injection: 2, 10 mg/ml, Granola and powder (in Canada) |
| Onset of action | 20-30 minutes after IM injection |
| Duration of action | Up to 6 hours |
| Interactions | α_1_ blockers, Vasodilators and other hypotensive drugs, Strychnine intoxication, Organophosphate intoxication, CNS suppressors, Aluminum salicylate (kaolin), Pectin, Bismuth, Anti-acids, Propranolol, Procaine, Phenytoin, Quinidine, Epinephrine, Opioids, Anesthetics, TCAs, Barbiturates |
| Adverse effects | Hypotension, Prolonged tranquilization, Constipation, Bradycardia, Prolonged false pregnancy, Increases risk of epilepsy and tetany |
| Pregnancy and lactation | Not recommended |

*Sedation is unreliable by use of Acepromazine alone.

| Buprenorphine Hydrochloride ^1,3,8–15^ | |
| --- | --- |
| Classification | Opioid analgesic (25-50 times more potent than Morphine) |
| Mechanism of action | Partial agonistic antinociception through μ- and nociception/orphanin FQ receptors;  agonist of κ- and δ-opioid receptor; inverse agonism for κ-opioid receptor and antagonism for δ-opioid receptor are suggested. |
| Indications | Analgesia (mild to moderate pain); sedation for minor procedures or premedication (usually in combination with acepromazine or dexmedetomidine). |
| Trade names | Temgesic, Buprenex, Buprecare, Buprenodale, Vetergesic, Buvidal, Sublocade |
| Pharmacokinetic | Metabolized by liver (prolongation of effect in animals with impaired liver function) |
| Dosage forms | Injectable: 1 ml ampules (0.3 mg/ml solution without preservative); 10 ml multidose vials (containing chlorocresol as preservative); prolonged-release formulations. |
| Onset of action | Relatively slow (>15 min): Slower than methadone. |
| Duration of action | Approximately 6 hours in rabbits and rodents |
| Interactions | Combining buprenorphine with other analgesics or anesthetic drugs, reduces the required doses of these drugs. |
| Adverse effects | Rarely occurs within prescribed doses, but include: respiratory suppression (depending on sex, body condition score, and leptin status), drug dependency, hear rate decrease. Adverse effects are reversible with naloxone (antidote).  The multidose formulation is unpalatable due to the taste of the preservative.  Pain at the intramuscular injection site of the multidose preparation. |
| Contraindication | Should not be used as a premedication if potent opioids would be used during surgery (buprenorphine as a partial agonist decreases the effects of the full agonists). |
| Pregnancy and lactation | Not recommended |

| Carprofen ^1,3,16–22^ | |
| --- | --- |
| Classification | Nonsteroidal anti-inflammatory drug (NSAID) |
| Mechanism of action | Inhibition of cyclooxygenase (COX) enzyme (preferentially COX2) and consequent decrease of inflammatory prostaglandin |
| Indications | Analgesia against pains of chemical origin; controlling postoperative inflammation;  controlling chronic inflammation (e.g., osteoarthritis or degenerative joint disease) |
| Trade names | Canidryl, Carprodyl, Dolagis, Rimadyl, Rimifin, Zinecarp |
| Pharmacokinetic | Metabolized by liver (prolongation of effect is expectable in mice with impaired liver function) |
| Dosage forms | Injectable: 50 mg/ml (Store in refrigerator); Oral: 20 mg, 50 mg, 100 mg tablets |
| Onset of action | Time to peak drug concentration:2 hours (PO, SC) |
| Duration of action | ~ 12 hours (SC administration) |
| Interactions | With other NSAIDs or glucocorticoids (more ulcerogenic when combined); Nephrotoxic agents (e.g., aminoglycosides such as gentamycin, streptomycin, and amikacin). |
| Adverse effects | Gastrointestinal signs such as bleeding (terminate the therapy if this remains more than 1–2 days). In some animals, adverse effects may result from a specific NSAID and not the other NSAIDs (Before shifting to another NSAID, a 1–2 week wash-out period should be considered); increase in plasma creatinine levels; increase of the myocardial structural disorganization; increased expression of pyrogenic pro-inflammatory cytokines TNF-α and IL-1β; Female mice appear to be more sensitive to carprofen during administration by the drinking water. |
| Contraindication | Hypovolemia, hypotension*, dehydration, gastrointestinal disease, blood clotting abnormalities, renal dysfunction, liver dysfunction**. |
| Pregnancy and lactation | Not recommend |

* If hypotension is expected during anesthesia, delay carprofen administration until full anesthetic recovery.

** Liver enzymes should be evaluated in long-term use of carprofen. An increased level of liver enzymes, may require cessation of the therapy with carprofen and shifting to other analgesics.

| Isoflurane ^1,2,23–35^ | |
| --- | --- |
| Classification | CNS agent, General anesthetic, Inhalation anesthetic |
| Mechanism of action | Volatile anesthetic, with not fully understood mechanism of action |
| Indication | Induction and maintenance of anesthesia |
| Trade names | Isocare, Isofane, IsoFlo, IsoVet, Vetflurane, Aerrane |
| Pharmacokinetic | Almost no metabolization in the liver (only 0.2%), fewer effect on the liver blood flow compared to halothane. |
| Dosage forms | 250 ml bottle |
| Onset of action | Rapid |
| Duration of action | Short duration of action (depending on its blood concentration) |
| Interactions | Concurrent use of opioid agonists, benzodiazepines and N2O reduces the required concentration of isoflurane for achieving surgical anesthesia. |
| Adverse effects | Dose-dependent hypotension (due to vasodilation), Dose dependent respiratory depression, arrhythmia (rare), Immunosuppression, Severe hypoglycemia in neonatal mice, Postoperative cognitive dysfunction (POCD), Induction of glucocorticoid production in mouse lymphoid organs, Dose dependent impaired auditory function, anterograde amnesia, Protection of blood-brain-barrier against cerebral extravasation, Activating leak Sodium conductance in the neurons of retro trapezoid nucleus. |
| Contraindication | Allergy and hypersensitivity to this drug |
| Pregnancy and lactation | Not recommended |

| Ketamine ^1,3,6,36–39^ | |
| --- | --- |
| Classification | CNS agent, General anesthetic, Dissociative anesthetic |
| Mechanism of action | Antagonizing the effect of the “glutamate excitatory neurotransmitter at *N*-methyl-d-aspartate (NMDA) receptors in the CNS”; May interact with opioid receptors by agonistic action at delta and kappa receptors and antagonizing mu receptors |
| Indication | Anesthesia or immobilization |
| Trade names | Anaestamine, Anesketin, Ketamidor, Ketaset injection, Ketavet, Narketan-10, Nimatek, Vetalar |
| Pharmacokinetic | Fast absorption, distributes to brain and metabolized by liver, one of its metabolites (Norketamine) has more antagonistic effects on NMDA receptors, elimination mostly by kidney |
| Dosage forms | Injectable: 50 mg/ml and 100 mg/ml solution |
| Onset of action | Rapid |
| Duration of action | Short acting (depending on blood concentration, usually 30 minutes or less) |
| Interactions | Alkaline (basic) solutions (the pH of ketamine solution should be maintained as acidic to preserve its stability and solubility) |
| Adverse effects | Mild increase of cardiac rate, cardiac output, and blood pressure; Cardiovascular depression; Arrhythmias in animals affected by shock or severe cardiovascular disease; Tachycardia following high dose intravenous injection; Respiratory depression; Abnormal behavior during recovery period; Drug accumulation and prolonged recovery; Pain at intramuscular injection site; Preservation of cranial nerve reflexes (e.g., the eyes remaining open during anesthesia); Hypertonicity and rigidity of skeletal muscles*; Anxiolytic-like effects; Anti-depressant effect; Cholinergic effects **(e.g., salivation, mydriasis, and vomiting. |
| Contraindication | Animals with high intracranial pressure; Epileptic patients; Animals with increased intraocular pressure. |
| Pregnancy and lactation | Not recommended |

*Co-administration of an alpha-2 adrenergic agonist and/or a benzodiazepine would ameliorate this effect. In these cases, reversal of the alpha-2 adrenergic agonist or benzodiazepine should be postponed for 45 min following ketamine administration.

**Prophylactic use of atropine may control this adverse effect.

| Xylazine Hydrochloride ^1–4,40–44^ | |
| --- | --- |
| Classification | Sedative, tranquilizer and analgesic |
| Mechanism of action | Agonistic effect on peripheral and central alpha-2 adrenoreceptors* |
| Indication | Usually used in combination with other drugs to generate surgical anesthesia; sedation; premedication; euthanasia. |
| Trade names | Chanazine, Nerfasin, Rompun, Sedaxylan, Virbaxyl, Xylacare, Xylapan |
| Pharmacokinetic | Fast absorption, distribution in the brain, metabolization by liver, and elimination by kidneys |
| Dosage forms | Injectable: 20 mg/ml solution |
| Onset of action | Rapid |
| Duration of action | Short acting (depending on blood concentration, usually 30 minutes or less) |
| Interactions | When using xylazine in an anesthetic combination, the dose of other anesthetics should be reduced; xylazine combined with opioid analgesics may significantly suppress CNS functions. |
| Adverse effects** | Sensitizes the myocardium to the effects of catecholamines, which may lead to cardiovascular dysfunction; mydriasis; decreased intraocular pressure; decreased insulin secretion leading to increased blood glucose levels; increased urine flow by suppression of anti-diuretic hormone secretion; xylazine overdose causes confusion, drowsiness, and loss of consciousness. |
| Contraindication | Animals with cardiovascular diseases; old animals; concurrent use of sympathomimetic medications; diabetic animals; increased intraocular pressure |
| Pregnancy and lactation | Not recommended (it may increase uterine motility and reduce fetal oxygenation) |

*Xylazine mainly stimulates alpha-1 adrenoreceptors and has less specificity for alpha-2 adrenoreceptor in comparison to medetomidine and dexmedetomidine. This lack of specificity may be the reason for narrower safety margin of xylazine compared to medetomidine and dexmedetomidine.

**Alpha-2 adrenoreceptors antagonists such as atipamezole and yohimbine may be used to reverse the effects of xylazine.

# References:

1. Hedley J, British Small Animal Veterinary Association. *BSAVA Small Animal Formulary Part B Part B*. 10th ed.; 2020. Accessed January 10, 2022. https://www.bsavalibrary.com/content/formulary/exotic-pets

2. Quesenberry KE, Orcutt CJ, Mans C, Carpenter JW. Ferrets Rabbits and Rodents clinical medicine and surgery. In: *Ferrets, Rabbits, and Rodents*. 4th ed. W.B. Saunders; 2020:iv. doi:10.1016/B978-0-323-48435-0.12001-5

3. Papich. *Saunders Handbook of Veterinary Drugs*. 2nd. ed, c2007.

4. Welberg LAM, Kinkead B, Thrivikraman KV, Huerkamp MJ, Nemeroff CB, Plotsky PM. Ketamine-xylazine-acepromazine anesthesia and postoperative recovery in rats. *J Am Assoc Lab Anim Sci*. 2006;45(2):13-20.

5. Gargiulo S, Greco A, Gramanzini M, et al. Mice Anesthesia. *Ilar J*. 2012;53(1):E55-:1-13.

6. Arras M, Autenried P, Rettich A, Spaeni D, Rülicke T. Optimization of intraperitoneal injection anesthesia in mice: drugs, dosages, adverse effects, and anesthesia depth. *Comp Med*. 2001;51(5):443-456.

7. Maddison JE, Page SW, Church DB. *Small Animal Clinical Pharmacology*. Vol 5. Elsevier Health Sciences; 2008.

8. Grinnell SG, Ansonoff M, Marrone GF, et al. Mediation of buprenorphine analgesia by a combination of traditional and truncated mu opioid receptor splice variants. *Synapse*. 2016;70(10):395-407. doi:10.1002/syn.21914

9. Navarro K, Jampachaisri K, Huss M, Pacharinsak C. Lipid bound extended release buprenorphine (high and low doses) and sustained release buprenorphine effectively attenuate post-operative hypersensitivity in an incisional pain model in mice (Mus musculus). *Anim Models Exp Med*. 2021;4(2):129-137. doi:10.1002/ame2.12157

10. Angel C, Glovak ZT, Alami W, et al. Buprenorphine Depresses Respiratory Variability in Obese Mice with Altered Leptin Signaling. *Anesthesiology*. 2018;128(5):984-991. doi:10.1097/ALN.0000000000002073

11. Rudeck J, Vogl S, Heinl C, et al. Analgesic treatment with buprenorphine should be adapted to the mouse strain. *Pharmacol Biochem Behav*. 2020;191:172877. doi:10.1016/j.pbb.2020.172877

12. Kendall LV, Singh B, Bailey AL, et al. Pharmacokinetics and Efficacy of a Long-lasting, Highly Concentrated Buprenorphine Solution in Mice. *J Am Assoc Lab Anim Sci*. 2021;60(1):64-71. doi:10.30802/AALAS-JAALAS-20-000049

13. Glovak ZT, Angel C, O’Brien CB, Baghdoyan HA, Lydic R. Buprenorphine differentially alters breathing among four congenic mouse lines as a function of dose, sex, and leptin status. *Respir Physiol Neurobiol*. 2022;297:103834. doi:10.1016/j.resp.2021.103834

14. Browne CA, Falcon E, Robinson SA, Berton O, Lucki I. Reversal of Stress-Induced Social Interaction Deficits by Buprenorphine. *Int J Neuropsychopharmacol*. 2018;21(2):164-174. doi:10.1093/ijnp/pyx079

15. Coutens B, Derreumaux C, Labaste F, et al. Efficacy of multimodal analgesic treatment of severe traumatic acute pain in mice pretreated with chronic high dose of buprenorphine inducing mechanical allodynia. *Eur J Pharmacol*. 2020;875:172884. doi:10.1016/j.ejphar.2019.172884

16. Kendall LV, Bailey AL, Singh B, McGee W. Toxic Effects of High-dose Meloxicam and Carprofen on Female CD1 Mice. *J Am Assoc Lab Anim Sci*. 2022;61(1):75-80. doi:10.30802/AALAS-JAALAS-21-000071

17. Krishnan V, Booker D, Cunningham G, et al. Pretreatment of carprofen impaired initiation of inflammatory- and overlapping resolution response and promoted cardiorenal syndrome in heart failure. *Life Sci*. 2019;218:224-232. doi:10.1016/j.lfs.2018.12.048

18. Halade GV, Kain V, Wright GM, Jadapalli JK. Subacute treatment of carprofen facilitate splenocardiac resolution deficit in cardiac injury. *J Leukoc Biol*. 2018;104(6):1173-1186. doi:10.1002/JLB.3A0618-223R

19. Cho C, Michailidis V, Lecker I, et al. Evaluating analgesic efficacy and administration route following craniotomy in mice using the grimace scale. *Sci Rep*. 2019;9(1):359. doi:10.1038/s41598-018-36897-w

20. Lindsey ML, Brunt KR, Kirk JA, et al. Guidelines for in vivo mouse models of myocardial infarction. *Am J Physiol-Heart Circ Physiol*. 2021;321(6):H1056-H1073. doi:10.1152/ajpheart.00459.2021

21. Ingrao JC, Johnson R, Tor E, Gu Y, Litman M, Turner PV. Aqueous stability and oral pharmacokinetics of meloxicam and carprofen in male C57BL/6 mice. *J Am Assoc Lab Anim Sci JAALAS*. 2013;52(5):553-559.

22. Foley PL, Kendall LV, Turner PV. Clinical Management of Pain in Rodents. *Comp Med*. 2019;69(6):468-489. doi:10.30802/AALAS-CM-19-000048

23. Greenfield EA. Administering Anesthesia to Mice, Rats, and Hamsters. *Cold Spring Harb Protoc*. 2019;2019(6):pdb.prot100198. doi:10.1101/pdb.prot100198

24. Anesthesia with sevoflurane or isoflurane induces severe hypoglycemia in neonatal mice. Accessed January 22, 2022. https://journals.plos.org/plosone/article?id=10.1371/journal.pone.0231090

25. Song J, Chu S, Cui Y, et al. Circadian rhythm resynchronization improved isoflurane-induced cognitive dysfunction in aged mice. *Exp Neurol*. 2018;306:45-54. doi:10.1016/j.expneurol.2018.04.009

26. Marquardt N, Feja M, Hünigen H, et al. Euthanasia of laboratory mice: Are isoflurane and sevoflurane real alternatives to carbon dioxide? *PLOS ONE*. 2018;13(9):e0203793. doi:10.1371/journal.pone.0203793

27. Friese MB, Nathan M, Culley DJ, Crosby G. Isoflurane anesthesia impairs the expression of immune neuromodulators in the hippocampus of aged mice. *PLOS ONE*. 2018;13(12):e0209283. doi:10.1371/journal.pone.0209283

28. Poon KS, Pan YL, Liao KH, et al. Isoflurane attenuates carbogen-induced blood–brain barrier disruption independent of body temperature in mice and rats. *NeuroReport*. 2020;31(2):118-124. doi:10.1097/WNR.0000000000001390

29. Zhang SS, Tian YH, Jin SJ, et al. Isoflurane produces antidepressant effects inducing BDNF-TrkB signaling in CUMS mice. *Psychopharmacology (Berl)*. 2019;236(11):3301-3315. doi:10.1007/s00213-019-05287-z

30. Pi Z, Lin H, Yang J. Isoflurane reduces pain and inhibits apoptosis of myocardial cells through the phosphoinositide 3-kinase/protein kinase B signaling pathway in mice during cardiac surgery. *Mol Med Rep*. 2018;17(5):6497-6505. doi:10.3892/mmr.2018.8642

31. Hamden JE, Salehzadeh M, Gray KM, Forys BJ, Soma KK. Isoflurane stress induces glucocorticoid production in mouse lymphoid organs. *J Endocrinol*. 2021;251(2):137-148. doi:10.1530/JOE-21-0154

32. Zhao W, Zhao S, Zhu T, et al. Isoflurane Suppresses Hippocampal High-frequency Ripples by Differentially Modulating Pyramidal Neurons and Interneurons in Mice. *Anesthesiology*. 2021;135(1):122-135. doi:10.1097/ALN.0000000000003803

33. Yang Y, Ou M, Liu J, et al. Volatile Anesthetics Activate a Leak Sodium Conductance in Retrotrapezoid Nucleus Neurons to Maintain Breathing during Anesthesia in Mice. *Anesthesiology*. 2020;133(4):824-838. doi:10.1097/ALN.0000000000003493

34. Sheppard AM, Zhao DL, Salvi R. Isoflurane anesthesia suppresses distortion product otoacoustic emissions in rats. *J Otol*. 2018;13(2):59-64. doi:10.1016/j.joto.2018.03.002

35. Isoflurane Veterinary - FDA prescribing information, side effects and uses. Drugs.com. Accessed April 21, 2022. https://www.drugs.com/pro/isoflurane-veterinary.html

36. He S, Atkinson C, Qiao F, Chen X, Tomlinson S. Ketamine-xylazine-acepromazine compared with isoflurane for anesthesia during liver transplantation in rodents. *J Am Assoc Lab Anim Sci*. 2010;49(1):45-51.

37. Zanos P, Moaddel R, Morris PJ, et al. Ketamine and Ketamine Metabolite Pharmacology: Insights into Therapeutic Mechanisms. Witkin JM, ed. *Pharmacol Rev*. 2018;70(3):621-660. doi:10.1124/pr.117.015198

38. Wei Y, Chang L, Hashimoto K. A historical review of antidepressant effects of ketamine and its enantiomers. *Pharmacol Biochem Behav*. 2020;190:172870. doi:10.1016/j.pbb.2020.172870

39. Rosenbaum SB, Gupta V, Palacios JL. Ketamine. In: *StatPearls*. StatPearls Publishing; 2022. Accessed February 14, 2022. http://www.ncbi.nlm.nih.gov/books/NBK470357/

40. Meyer GMJ, Meyer MR, Mischo B, Schofer O, Maurer HH. Case report of accidental poisoning with the tranquilizer xylazine and the anesthetic ketamine confirmed by qualitative and quantitative toxicological analysis using GC-MS and LC-MSn. *Drug Test Anal*. 2013;5(9-10):785-789. doi:10.1002/dta.1475

41. Mohammed abd el-nasser, Al-Hozab A, Al-Shaheen T. Effects of Diazepam and Xylazine on Changes of Blood Oxygen and Glucose Levels in Mice. *Adv Anim Vet Sci*. 2018;6. doi:10.17582/journal.aavs/2018/6.3.121.127

42. Jamal MA, Ahmed AM, Tahir M, et al. Safety and efficacy of ketamine xylazine along with atropine anesthesia in BALB/c mice. *Braz J Pharm Sci*. 2019;55:e17231. doi:10.1590/s2175-97902019000317231

43. Shaban KA, Alzubaidy MH, Faris GA. Evaluation of the antinociceptive effect of xylazine and it’s interaction with metoclopramide in the acute pain model in mice. *Iraqi J Vet Sci*. 2020;34(2):383-388.

44. Schoell AR, Heyde BR, Weir DE, Chiang PC, Hu Y, Tung DK. Euthanasia Method for Mice in Rapid Time-Course Pulmonary Pharmacokinetic Studies. *J Am Assoc Lab Anim Sci*. 2009;48(5):506-511.
